# Effectiveness of Peer-Led Interventions in Improving the Dietary Behavior of Adolescents in Low- and Middle-Income Countries: A Systematic Review

**DOI:** 10.1093/nutrit/nuaf037

**Published:** 2025-03-27

**Authors:** Daniale T Ekubagewargies, Faruk Ahmed, Patricia Lee

**Affiliations:** Public Health, School of Medicine and Dentistry, Griffith University, Gold Coast, QLD 4222, Australia; Public Health, School of Medicine and Dentistry, Griffith University, Gold Coast, QLD 4222, Australia; Public Health, School of Medicine and Dentistry, Griffith University, Gold Coast, QLD 4222, Australia

**Keywords:** peer-led interventions, dietary behaviors, adolescents, nutritional education, customization, low- and middle-income countries

## Abstract

**Context:**

Adequate nutrition is crucial during adolescence due to significant physical, mental, and emotional changes, yet in many adolescents poor dietary behaviors lead to inadequate nutrient intake and increased health risks. Peer-led interventions have shown promise in improving these behaviors. Thus, synthesizing evidence from primary studies is crucial to enhance their effectiveness and policy implications.

**Objective:**

In this review we synthesized evidence on the effectiveness of peer-led interventions in improving the dietary behavior of adolescents in low and middle-income countries (LMICs).

**Data Sources:**

We searched thePubMed, CINAHL, PsycINFO, ERIC, Cochrane Library, and SCOPUS databases for studies on peer-led interventions among adolescents aged 10-19 years. No specific publication timeframe was set for the search. Studies lacking quantitative outcome measures were excluded.

**Data Extraction:**

Of the 3177 records initially identified, 8 studies were included. Study quality was assessed by use of Joanna Briggs Institute quality appraisal tools. Data extraction involved capturing study characteristics, intervention components, outcomes, and key findings.

**Data Analysis:**

The studies were conducted in the regions of Sub-Saharan Africa, the Middle East, Southeast Asia, and South Asia, with intervention durations ranging from 3 weeks to 3 years. Interventions included peer leaders facilitating group discussions, making posters, and providing practical demonstrations. Most studies reported improvements in dietary intake such as increases in fruit and vegetable consumption and reductions in unhealthy snack intake following the intervention. Knowledge and attitudes toward healthy dietary behavior also improved. None of the studies included explicit detail involving adolescents in the initial design of interventions. Multicomponent interventions and longer durations were more successful.

**Conclusion:**

Peer-led interventions effectively improved the dietary behaviors of adolescents in LMICs. The findings of this review underscore the importance of multicomponent strategies and longer intervention durations. Involving adolescents in program design is recommended to enhance the relevance and impact of interventions. Researchers should identify the most effective intervention components and delivery methods.

## INTRODUCTION

Adolescence is the time when substantial changes in physical, mental, and emotional spheres are observed; as a result, nutritional requirements increase significantly.^1^ A nutritionally adequate, safe, and good-quality diet is essential for adolescent health, school performance, and future employment.^2^ However, the dietary behaviors of adolescents are characterized by poor diet patterns such as frequent snacking, fast-food consumption, and meal skipping, and thus many adolescents do not meet the recommended dietary intake for different nutrients^3^^,^^4^ and are at risk of developing nutrition-related health problems.^5^^,^^6^ The critical implications of poor dietary behavior extend beyond immediate health risks, predicting dietary behavior in adult years.^7^ Adolescents in low- and middle-income countries (LMICs) face a range of nutrition challenges, including thinness, growth stunting, overweight and obesity, anemia, and other micronutrient deficiencies.^8^^,^^9^ Addressing these challenges may involve nutritional intervention, with purposefully planned actions intended to positively change a nutrition-related behavior, environmental condition, or aspect of health status.^10^^,^^11^

Adolescence, marked by increased autonomy, significantly influences health-related behaviors like dietary habits.^3^^,^^12^ In LMICs, these changes are often characterized by increased consumption of processed and sugary foods, meal skipping (particularly breakfast), and eating away from home.^13^ Peers of the same age become crucial influencers during this period and exert an influence on each other’s dietary behavior.^14^^,^^15^ Capitalizing on the concept of peer pressure, interventions leveraging peer-to-peer interactions, known as peer-led interventions, have proven effective in inducing behavior change.^7^ Such interventions facilitated by peers are particularly potent, as peers are seen as more credible and relatable sources of information than adults.^16^

Knowledge is a critical determinant of behavior change, although it may not always be sufficient on its own to produce significant improvements in dietary practice. Research has shown that knowledge regarding nutrition can positively influence dietary practices, yet this relationship is often mediated by other intrapersonal and environmental factors. Story et al.^17^ argued that nutrition knowledge is one of many intrapersonal factors that contribute to changes in eating behavior.

While knowledge can be a powerful tool in shaping positive attitudes and encouraging healthy dietary choices,^18^^,^^19^ it does not always directly translate into behavior change. Factors such as motivation, environmental barriers, and social influences can complicate this relationship. Therefore, when evaluating the effectiveness of nutrition interventions, it is crucial to consider the complex interplay between knowledge, attitudes, and the broader social and environmental context.

Socio-cognitive models such as Bandura’s Social Cognitive Theory,^20^ Rosenstock’s Health Belief Model,^21^ and Ajzen’s Theory of Planned Behavior,^22^ have been widely applied in the development of nutrition education interventions. These models focus on the role of beliefs, social norms, and perceived behavioral control in shaping dietary decisions, which makes them highly relevant for designing and evaluating peer-led interventions aimed at adolescents. Grounding interventions with psychological theories helps to clarify how they foster long-term dietary behavior changes, making them better suited to meet the needs of the adolescent population.^7^ Moreover, the influence of environmental factors (availability, affordability, and social support) is acknowledged within these models, further enhancing the relevance of these frameworks in designing comprehensive interventions.

Understanding behavior change interventions is crucial to identifying the mechanisms driving change. Identification of these mechanisms helps in improving intervention effectiveness and refining strategies to improve health outcomes.^23^ Several frameworks are used for this purpose, each offering unique insights into how behavior change interventions are designed, implemented, and assessed. The Behaviour Change Wheel (BCW) provides a comprehensive structure for categorizing intervention functions and understanding behavior change.^23^ The Theoretical Domains Framework (TDF) synthesises key constructs from multiple theories to help identify barriers and facilitators of change.^24^ While the behaviour change techniques taxonomy (v1) offers a systematic classification of specific techniques, such as self-monitoring and goal setting, used in interventions,^25^ Intervention Mapping provides a step-by-step approach to designing health promotion programs by integrating theory and evidence into the planning and evaluation process.^26^

From the ages of 9-10 years onward, family- or home-based interventions targeting the behavior of children and adolescents may become less effective than school- or community-based interventions^27^ for 2 reasons: First, as children grow older, the most crucial relationship they form is with their peers, not family members.^28^^,^^29^ Second, adolescents spend a substantial amount of time at school, a critical period for habit formation,^30^^,^^31^ making schools an ideal setting for fostering healthy habits.^32^^,^^33^ Given that school environments are more malleable than home- or community-based factors,^29^ interventions in schools can significantly impact adolescents’ dietary habits.^34^ Recognizing the cost-effectiveness of school-based interventions^35^ and the potential for substantial returns on investment in LMICs,^36^ prioritizing health-promoting initiatives in schools is crucial from a public health standpoint.

Peer-led interventions have been shown to improve the dietary behaviors of adolescents, including increased fruit and vegetable intake, increased breakfast consumption, and enhanced knowledge and attitudes toward healthy eating. Reviews by Yip et al.,^7^ Vangeepuram et al.,^37^ and Frerichs et al.^38^ demonstrated these positive outcomes in high-income countries. However, these studies were primarily conducted in socio-economic and environmental contexts distinct from those in LMICs, limiting their relevance to these regions.

A significant gap exists in our understanding of how peer-led interventions perform in LMICs, where the dual burden of malnutrition—undernutrition and overnutrition—creates unique challenges.^39^ In LMICs, the intake of processed foods is rising, while nutrient deficiencies and undernutrition remain prevalent.^39^ These diverse cultural and socioeconomic contexts require that peer-led interventions be tailored to local needs, yet to our knowledge no previous reviews have addressed the adaptability of these interventions across various settings.

In this systematic review we aimed to evaluate how peer-led interventions can be contextualized to meet the specific needs of adolescents in LMICs. The insights gained are invaluable for designing effective, sustainable, and culturally relevant interventions to improve adolescent dietary behavior and inform nutrition policies in LMICs. Thus, the purpose of this systematic review is to answer the research question: “What is the effect of peer-led, school-based nutritional interventions on the dietary behavior of adolescents in LMICs?”

## METHODS

The reporting style of this review is based on the PRISMA (Preferred Reporting Items for Systematic Reviews and Meta-analysis) guidelines.^40^ In this review we examined intervention studies in LMICs utilizing peer-led approaches to enhance healthy dietary behaviors among adolescents, encompassing areas such as increased fruit and vegetable consumption, reduced sugar-sweetened beverage intake, and improved overall diet quality. We also assessed the impact of these interventions on adolescents’ knowledge and attitudes, recognizing the importance of knowledge as a precursor to positive attitudes and improved behavior.^18^^,^^19^

### Inclusion Criteria

Studies selected for inclusion had the following characteristics:

- Studies conducted in LMICs (Gross National Income per capita below $13 205). As of the 2023 fiscal year, the World Bank has classified countries according to individual annual incomes as follows: low income (less than $1085), lower-middle income ($1086 to $4255), and upper-middle income ($4256 to $13 205).^41^
- Peer-reviewed original studies.
- Included an intervention component as the main research method.
- Included a nutrition intervention that was led by peers (same age or older).
- Took place in a school setting (primary school or high school).
- Investigated outcomes for school-aged youth (10-19 years old). Studies involving children in primary or elementary school were included in the review only if a subgroup analysis specifically for adolescents was also provided.
- Reported quantitative results or had a quantitative component if it was a mixed-method study.
- Published in the English language.
- No limit was set on the publication date.

### Exclusion Criteria

Studies with the following characteristics were excluded:

- The intervention was led exclusively by adults or professionals.
- Included children younger than 10 years or older than 19 years without subgroup analysis.
- Purely qualitative studies.
- Additional research reports from the studies that were already included.

### Information Sources

The following databases were searched: PubMed, CINAHL, PsycINFO, ERIC, SCOPUS, and the Cochrane Library. Hand-searching of reference lists of previously published systematic reviews on the topic was done. Also, reference lists of included studies were hand searched.

### Search Method

All of the available literature on peer-led nutritional intervention in LMICs was screened using study titles and abstracts based on the criteria listed above. The systematic search was commenced in December 2022 and the final search was conducted in April 2024. The search strategy was crafted to be comprehensive, focusing on 4 main components—population (such as adolescents and teenagers), intervention (such as peer-led nutrition interventions), setting (such as schools and educational settings), and outcome (such as dietary behavior and dietary intake). Keywords were used in the search and included “adolescent,” “dietary behavior,” “nutritional intervention,” “peer-led,” and “schools” (see Table 1).

**Table 1.** PICOS criteria for study inclusion.

Parameter	Criterion
Participants	School adolescents aged 10-19 y in LMICs
Interventions/exposure	Nutrition education through a peer-led approach
Comparisons	Adolescents who did not participate in peer-led interventions
Outcomes	Improvements in dietary intake, enhanced knowledge and attitudes toward healthy dietary behaviors, and improved self-efficacy
Study design	RCTs and quasi-experimental designs

Abbreviation: LMICs, low- and middle-income countries; RCT, randomized controlled trial.

### Study Selection

During our initial search, we looked for both qualitative and quantitative studies, keeping the screening stage open to all languages, dates, and research designs. However, certain limitations were later applied using the inclusion and exclusion criteria. During the development of search strategies, a university librarian with expertise in systematic review research was consulted. The primary author (D.T.E.) screened the titles and abstracts of the articles identified by the search to determine eligibility while a second researcher (A.T.K.) independently reviewed the search results and verified the inclusion and exclusion criteria. Then, full-text articles were obtained for studies that were deemed eligible and assessed using predetermined inclusion and exclusion criteria.

### Assessment of Risk of Bias Within Studies

The quality of the included studies was assessed using the Joanna Briggs Institute (JBI) quality appraisal tools by D.T.E. and A.T.K. tThese appraisal tools offer a robust, validated, and user-friendly approach tailored to healthcare research, including interventions,^42^^,^^43^ making these tools an excellent choice for appraising the quality of studies included in our systematic review. We used randomized controlled trials (RCTs) and quasi-experimental design checklists to accommodate the type of interventions used in the original studies. The quality appraisal tool for the quasi-experimental design included components of the temporal relationship of variables, the similarity among compared groups, the similarity of treatment received other than the intervention of interest, control groups, multiple measures, complete follow-up, measurement of outcomes among compared groups, reliability of outcome measures, and appropriate statistical analysis. Likewise, the quality appraisal tool for the RCT design included components of randomization; concealment of allocation; blinding of participants, personnel, and outcome; similarity of groups at baseline; similarity of treatment received other than the intervention of interest; completeness of follow-up; appropriateness of analysis for randomization of groups; similarity of outcome measures for different groups; reliability of outcome measures; appropriateness of statistical analyses; and appropriateness of trial design. All of the studies were found to be of moderate to high quality, prompting for inclusion.

### Data Collection Process

Data were extracted from the included studies by use of a standardized data extraction form developed in Microsoft Excel. The data collected included information on the names of authors, study year, follow-up period, study objectives, participant demographics, interventions and comparators used, outcomes measured, study design, and key findings.

The results of the included studies were synthesized using narrative synthesis (emphasizing quantitative findings), with a focus on the effectiveness of peer-led interventions in promoting healthy dietary behaviors among adolescents in LMICs.

### Outcome Measures

Outcome measures included changes in any of the following outcomes: knowledge about healthy dietary behaviors; self-efficacy or attitudes toward healthy dietary behavior; and changes in dietary intakes, such as an increase in consumption of fruits and vegetables and a decrease in consumption of sugar-sweetened beverages, salty snacks, or processed foods.

## RESULTS

### Study Selection

**Figure 1.**
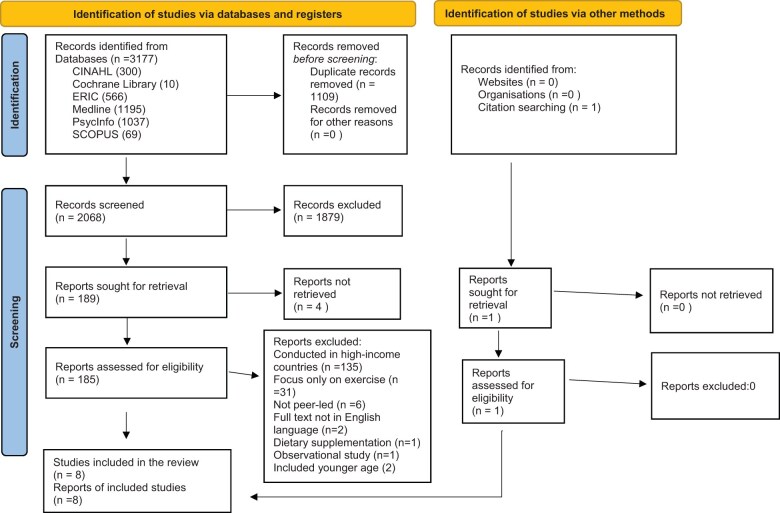
Flow diagram of study selection for inclusion in the review.

After a comprehensive database literature search, 3177 records were initially identified. After duplicates were removed (*n* = 1109), 2068 records were screened. Out of these, 1879 records were excluded, and 189 full-text articles were sought for access to full text. Four of these articles were not accessed in full length. Of the 185 fully accessed articles, the following were removed due to various reasons: studies conducted in high-income countries (*n* = 135), outcomes focusing only on physical activity or sedentary behavior (*n* = 31), interventions that were not peer led (*n* = 6), article full text not available in the English language (*n* = 2), focus on dietary supplementation (*n* = 1), observational study (*n* = 1), or included younger age participants without showing age range (*n* = 2). After these articles were removed, the 7 remaining articles were included, with the addition of 1 article found from citation searching. Thus, a total of 8 articles were found to be eligible for inclusion in the systematic review (Figure 1).

As indicated in Table 2,^44–51^ of the 8 included studies, 3 were conducted in Sub-Saharan Africa (Dargie et al.^44^ and Tamiru et al.^45^^,^^46^), 2 in the Middle East (Farrokhmanesh et al.^47^ and Maatoug et al.^48^), 2 in Southeast Asia (Sharif Ishak et al.^49^ and Thi Nguyen et al.^50^), and 1 in South Asia (Singhal et al.^51^). All of the included studies were published between the years 2010 and 2022. The study designs varied between quasi-experimental (*n* = 4) and RCTs (*n* = 4). Durations of the studied interventions ranged from 3 weeks to 3 years. All except 2 studies measured outcome variables twice—the remaining 2 studies measured outcome variables at 3 time points. All studies in this review assessed changes in dietary intake, while 3 studies investigated how interventions in adolescents could boost their knowledge of nutrition and dietary habits. Three studies examined changes in attitudes toward a healthy diet, while only 1 study looked at changes in self-esteem.

**Table 2.** Peer-led nutrition intervention programs for adolescents in LMICs.

Author and publication year	Year of study	Region/continent/country	Sample size	Design	Follow-up period	Repeated measures	Intervention	Dependent variable(s)	Key findings
Dargie et al. (2018)^44^	2016	East Africa/Africa/Ethiopia	202 students from 4 schools	RCT	6 months	2 (baseline and post-intervention)	Peer-led pulse food messages through brief nutrition education, peer group discussions, pamphlets, posters, and practical demonstrations	Knowledge, attitude, practice of pulse consumption, and nutritional status	Significant changes in knowledge, attitude, and practice in mean scores about pulse preparation and consumption, and reduced thinness among the intervention group
Farrokhmanesh et al. (2018)^47^	2014-15	Middle East/Asia/Iran	6 schools in 2 districts with an overall sample size of 188	RCT	3 weeks	2 (baseline and postintervention)	Child-to-child teaching and health educator–to-child teaching about food frequency and content of food	Nutritional intake	Child-to-child teaching was better compared to the performance of health educators and control group in improving nutritional behavior
Maatoug et al. (2015)^48^	2009/2010-2013/2014	Middle East/Africa/Tunisia	4275 students from 15 selected middle schools	QED	3 y	2 (baseline and postintervention)	Influential peer-led education on adopting healthy behaviors such as increasing daily fruit and vegetable consumption	Dietary intake and physical activity	Significant improvement in daily fruit and vegetable consumption and decreased intake of fried food and fast-food among intervention group compared to control
Sharif Ishak et al. (2020)^49^	2016-2017	Southeast Asia/Asia/Malaysia	201 students from 2 randomly selected schools	QED	3 mo	3 (baseline, postintervention, and after 3 mo)	A peer-led, school-based health promotion program that teaches cognitive and behavioral skills	Healthy eating (no meal skipping, decrease fast food consumption and consumption of food away from home, and increase family meal consumption), positive body image	Intervention was effective in improving knowledge supporting healthy eating and lifestyle but not dietary intake or attitude
Singhal et al. (2010)^51^	2008-2009	South Asia/Asia/India	209 students from 2 schools	RCT	6 mo	2 (baseline and postintervention)	Multicomponent intervention that includes dissemination of health-related information through lectures and focused group discussions, change in school policy, parent involvement and training of peer leaders to supplement the intervention effect	Knowledge, attitude, and intake of healthy diet; exercise, and anthropometry	A significant increase in nutrition-related knowledge, fruit intake as well as reduced consumption of fizzy drinks among the intervention group
Tamiu et al. ^a^ (2016)^45^	2013-2014	East Africa/Africa/Ethiopia	1000 students from 4 primary schools	QED	8 mo	3 (baseline, 6 mo, and 9 mo	Peer-led nutrition education sessions and establishment of home gardens	Dietary diversity	Significant improvement in dietary diversity of adolescents in intervention schools
Tamiru et al.^a^ (2017)^46^	2013-2014	East Africa/Africa/Ethiopia	1000 students from 4 primary schools from both rural and urban settings	QED	8 mo	3 (baseline, 6 mo, and 9 mo	Peer-led nutrition education sessions, health promotion through school media and health clubs	Optimal dietary practice and nutritional status	A significant improvement of animal source dietary intake in intervention schools
Thi Nguyen et al. (2022)^50^	2018-2019	South-East Asia/Asia/Vietnam	263 grade 6 students from 4 schools	RCT	9 mo	2 (baseline and postintervention)	Peer education and support (personal diet record diary monitoring, a classroom merit board), and school awards	Sedentary behaviors and dietary intake	Intervention improved dietary intake (reduced total energy intake, fat, carbohydrate, and sweet foods) and reduced sedentary time

Abbreviations: QED, quasi-experimental design; RCT, randomized controlled trial.

a Tamiru et al.^45^ and Tamiru et al.^46^ used the same setting. However, the 2 articles differ in included intervention components as well as outcome measures.

This systematic review revealed that peer-led interventions for disseminating messages among adolescents used a variety of approaches. All studies incorporated direct communication between peer leaders and students, but many supplemented this information with additional strategies. These included the use of school media and health clubs, posters, flyers, and organized school events to reinforce messages. Some studies, like those by Tamiru et al.^45^^,^^46^ engaged parents and the community, while others, such as Maatoug et al.^48^ and Farrokhmanesh et al.^47^ utilized interactive methods like demonstrations and educational events. Programs like the EPaL initiative by Sharif Ishak et al.^49^ and the intervention by Thi Nguyen et al.^50^ incorporated structured sessions and cognitive and behavioral skills training.

#### Intervention Characteristics

The included studies employed a variety of peer-led interventions targeting changes in dietary behavior. While all of the interventions shared a core peer-led component, they differed significantly in terms of design, delivery methods, duration, and supplementary elements such as family involvement, media use, and community engagement.

To evaluate the peer-led interventions, we applied the BCW framework, which categorizes interventions by functions like education, persuasion, and incentivization, enabling assessment of the mechanisms driving behavior change.^23^

In the education component of BCW, interventions focus on imparting knowledge and awareness to influence attitudes and behaviors. In this review, studies used varied approaches for education, such as peer-led discussions, cooking demonstrations, multimedia tools, role-playing, and family engagement.^44–51^

Incentivization includes using rewards, reinforcements, or recognition to encourage healthy dietary practices; only Thi Nguyen et al.^50^ implemented incentivization through merit boards and diet diaries, through which students received rewards for meeting dietary goals.

Enablement provides participants with tools, skills, or environmental changes that allow them to take control of their dietary behaviors. Studies used cooking demonstrations to equip participants with practical skills,^44^ hands-on gardening activities to empower students to grow and consume diverse foods,^45^^,^^46^ and the use of diet diaries and self-monitoring tools to enable participants to track their food consumption.^50^

Modeling refers to the demonstration of healthy behaviors by peers or role models, which others can observe and replicate. The studies reported here used role-playing activities^44^^,^^51^ and encouraged participants to emulate healthy dietary behaviors,^45^^,^^46^^,^^50^ allowing participants to observe and replicate these activities.

Environmental restructuring within the BCW framework involves altering the physical or social environment to make healthy behaviors more accessible. In this review, environmental restructuring was achieved by involving family members,^45^^,^^46^^,^^50^ establishing school health clubs,^46^ and introducing healthier food options in school canteens.^51^

In this review, 2 studies explicitly incorporated psychological frameworks. In the intervention by Dargie et al.,^44^ application of Ajzen’s Theory of Planned Behavior was used to target attitudes, subjective norms, and perceived behavioral control. Students were educated on the nutritional benefits of pulses (beans, peas, and lentils) to foster positive attitudes and placed in a socially supportive environment to encourage pulse consumption. Practical cooking demonstrations were provided to enhance perceived behavior control, equipping students with the skills and confidence to incorporate pulses into their diets independently. In contrast, Thi Nguyen et al.^50^ promoted behavior change through Bandura’s Social Cognitive Theory by integrating observational learning, self-regulation, and reinforcement. Students observed healthy behaviors from peers, used diet diaries for self-monitoring, and were motivated by rewards through merit boards and school awards, all of which helped sustain long-term behavior change.

Students who participated in interventions such as those used by Tamiru et al.^45^^,^^46^ and Singhal et al.,^51^ which incorporated multicomponent elements alongside peer-led education—such as family involvement, community engagement, and environmental restructuring—showed more sustained behavior change than students who participated in single-component approaches. Similarly, interventions with longer durations, such as those by Maatoug et al.^48^ (3 years), Thi Nguyen et al.^50^ (9 months), and Tamiru et al.^45^^,^^46^ (8 months) led to more favorable changes in dietary behaviors compared to shorter interventions such as those reported by Farrokhmanesh et al.^47^ (3 weeks) and Sharif Ishak et al.^49^ (3 months).

In summary, the 8 studies included in this review demonstrated that peer-led interventions can effectively improve dietary behaviors among adolescents, particularly by increasing the consumption of fruits, vegetables, and animal-source foods, while reducing the intake of unhealthy foods. Most studies also reported positive changes in dietary knowledge and attitudes,^44^^,^^49^^,^^51^ with 1 study highlighting improvements in self-esteem.^51^ Multicomponent interventions and those incorporating behavioral change theories and longer duration were more successful in achieving sustained improvements.

### Intervention Effects on Outcome Variables

All studies showed participant improvements from intervention in at least 1 of the components of dietary behavior measures (ie, dietary intake, knowledge, attitude, and self-efficacy). A summary of findings for changes in different outcomes is presented below.

### Dietary Intake

In all of the studies included in this review, the investigators tried to address the effects of peer-led interventions on the dietary intake (frequency of intake, type of food consumed, or both) of adolescents.

In the study by Dargie and colleagues,^44^ an RCT with pre- and post-tests showed that the mean diet diversity score significantly improved in the intervention group from 3.68 to 7.79, while the control group’s practice score decreased slightly (from 4.02 to 3.69), resulting in a significant difference between the groups (*t* = 12.40, *P* < .001) after the six months intervention. The study by Tamiru and colleagues^45^ reported a minor yet noteworthy variation in dietary diversity between the intervention and control groups during the midline survey after 6 months of intervention. However, the end-line data revealed a substantial difference in dietary diversity intake between the intervention and control groups; adolescents who received dietary intervention were 55% more likely to consume a diversified diet (OR = 2.55, 95% (1.55, 3.50), *P* =  .01) than adolescents who were in control group.

The study by Farrokhmanesh et al.^47^ indicated that intervention successfully improved the dietary intake of adolescents. This RCT found that child-to-child approaches were effective in increasing the frequency of intake of healthy snacks and fruits while reducing the intake of high-calorie, unhealthy snacks compared to the control group (OR = 2.25, *P* = .01). Similarly, the study by Maatoug and colleagues^48^ concluded that by the end of 3 years of intervention, the intake of recommended fruits and vegetables significantly increased from 30.0% to 33.2% in the intervention group (*P* = .03). In contrast, the control group saw a significant decrease from 40.2% to 35.0% (*P* = .001). Additionally, the intake of fried food and fast food decreased in the intervention group although these changes were not statistically significant. Tamiru and colleagues^46^ reported in their study that the intervention group showed a notable increase in animal-source food consumption after intervention (*P* < .001), while for the control group this consumption remained significantly lower (adjusted odds ratio = 0.26; 95% CI: 0.16-0.42). In the study by Thi Nguyen et al.,^50^ when the authors compared the pre-and postintervention assessments within each group, the intervention group exhibited a noteworthy reduction in their intake of total energy (by 304 kcal/d), protein (by 15 g/d), fat (by 13 g/d), carbohydrates (by 39 g/d), and sweet foods (by 20 g/d) (*P* < .05 for all) after a 9-month follow-up period, while the control group did not experience any significant changes. In terms of fruit consumption, the intervention group did not alter their daily intake, but the control group showed a decrease of approximately 40 g/d. A study by Singhal and colleagues^51^ with multicomponent intervention showed improvements in healthy dietary intake and reduced intake of unhealthy dietary intake among adolescents in the intervention group compared to the control group. For example, the intervention group exhibited a notably higher percentage (32.8%) of children consuming 2 glasses of milk daily, in comparison to the control group, of whom only 7.8% exhibited the same behavior. Furthermore, 9.9% of children in the intervention group increased their weekly fresh fruit intake while the control group experienced a 6.5% decrease, although this idifference was not statistically significant. A significantly smaller percentage of children in the intervention group consumed fizzy drinks (15%, *P* < .001) compared to the control group (34%, *P* < .001), and fewer children in the intervention group consumed energy-dense, unhealthy foods like burgers, pizzas, and French fries (9%, *P* = .03) compared to the control group (16%, *P* = .03).

Different from the above findings, a study by Sharif Ishak and colleagues^49^ on the effect of peer-led intervention on dietary practice found no significant difference between the intervention and control groups in dietary practices after 3 months of intervention.

In summary, 7 out of the 8 included studies demonstrated the effectiveness of peer-led interventions in improving dietary intake among participants. These interventions improved the intake of healthy snacks^47^ and fruit and vegetables,^47^^,^^48^^,^^51^ dietary diversity,^44^^,^^45^ and animal-source foods.^51^^,^^46^ The study participants also reduced their intake of total energy, protein, fat, carbohydrates, and sweet foods^50^ and decreased their consumption of white bread, fizzy drinks, and energy-dense foods like burgers and pizzas.^48^^,^^51^

### Dietary Knowledge

Three studies investigated the effects of interventions in boosting knowledge of nutrition and dietary habits in adolescents, and findings in all 3 studies demonstrated that adolescent in the intervention groups showed improved knowledge levels.

The study by Singhal et al.^51^ investigated the impact of a controlled intervention on behavior modification among adolescents in North India. This study demonstrated that after a 6-month intervention, the intervention group showed notable increases in knowledge of simple and complex carbohydrates (*P* = .003), the concept of empty calories (*P* < .001), sources and negative effects of trans-fats (*P* < .001), high-fat milk products (*P* = .002), refined cereals (*P* = .003), the importance of dietary fiber (*P* = .02), and the causes and types of diabetes (*P* < .001). Similarly, the study by Sharif Ishak et al.^49^ found that the intervention improved student participant’s knowledge, reporting significantly higher knowledge of nutritional concepts related to healthy eating among the intervention group compared to the control group at both Post I (adjusted mean difference = 3.34; 95% CI: 0.99-5.69; *P* = .006) and Post II (adjusted mean difference = 2.82; 95% CI: 0.86-4.78; *P* = .005).

In the same vein, the intervention conducted by Dargie et al.^44^ revealed significant differences in the mean scores for knowledge regarding pulse preparation and consumption among adolescents. The mean (SD) knowledge score for the intervention group increased from 4.03 (1.49) at baseline to 9.04 (1.41) postintervention, while the control group showed little change, with the mean (SD) scores going from 4.18 (2.58) to 4.30 (2.19) (*P* < .001).

### Dietary Attitude

Three studies in this review addressed participant attitudes toward diet: Dargie et al.,^44^ Sharif Ishak et al.,^49^ and Singhal et al.^51^ The first study demonstrated significant changes in mean (SD) attitude scores in the intervention group, which increased from 4.06 (2.43) at baseline to 7.87 (1.43) post-intervention, while the attitudes in the control group remained relatively unchanged, with scores shifting slightly from 4.14 (2.53) to 4.10 (1.73) (*P* < .001).^44^ Likewise, participants in the study by Singhal and colleagues showed improvements in attitude scores. For example, there was a significant increase (15.1%; *P* = .03) in the proportion of children in the intervention group who believed that consuming deep-fried, high-calorie Indian junk food daily would be harmful to their health.^51^ However, the study by Sharif Ishak et al.^49^ did not yield evidence of a significant association with improvements in attitudes toward adopting healthy eating habits.

### Self-Esteem

The only study included in this review that tried to understand the effect of peer-led interventions on self-esteem is the study by Singhal et al.^51^ This study demonstrated a positive impact on body image and self-esteem in the adolescent participants, with no significant negative effects observed. The proportion of students who avoided clothes due to feeling overweight decreased by 9.4% in the intervention group compared to a 7.4% increase in the control group, although this difference was not statistically significant.

## DISCUSSION

This is the first systematic review to explore the effectiveness of peer-led interventions in changing the dietary behavior of adolescents in LMICs. The evidence found in this systematic review suggests that school-based nutrition interventions that are peer led or have peer-led components can be effective in improving the dietary behaviors of adolescents across different geographic locations and study designs. Many health-related interventions used peer-led approaches and showed successful results in outcomes of interest ranging from improved nutrition to improved physical activity^52^ and effected positive change in HIV prevention^53^ and reductions in tobacco, alcohol, and drug use.^54^^,^^55^

### Dietary Intake

This systematic review demonstrated that peer-led interventions can effectively improve the dietary intake of adolescents. This finding aligns with the results of a previous systematic review by Yip and colleagues.^7^ Their review of studies conducted in Canada and the United States reported that 85% of included studies assessing diet or dietary intake changes found immediate improvements following peer-led interventions, which included increased consumption of fruits and vegetables, reduced intake of sugar-sweetened beverages, and decreased fat intake, indicating a positive shift toward healthier eating habits.^7^

Increased consumption of animal-source foods reported in the present review offers significant benefits for adolescent growth and development, as these foods are associated with improved micronutrient status, cognitive function, and motor skills.^56^^,^^57^ These findings suggest that peer-led nutrition interventions can play a crucial role in addressing nutritional deficiencies in LMICs where these issues associated with these deficiencies are prevalent. Peer-led interventions have also shown success in reducing the consumption of unhealthy foods. The present review revealed reductions in the consumption of sugar-sweetened foods, fizzy drinks, and energy-dense foods among adolescents after interventions. These findings are in alignment with those of a scoping review by Vangeepuram et al.,^37^ which found that peer-led interventions effectively reduced the intake of sugar-sweetened beverages and increased consumption of low-fat foods. This result is particularly encouraging given the negative health consequences associated with excessive sugar intake, such as weight gain, tooth decay, and insufficient intake of essential vitamins and minerals.^58^ Moreover, the reduction in soft drink consumption during adolescence is crucial, as it helps mitigate the risk of decreased milk consumption, which could lead to deficiencies in calcium and vitamin D—both vital for optimal bone mass.^59^

In this review, an increase in fruit and vegetable intake was a significant improvement in dietary behavior. This finding aligns with 2 reviews from high-income countries,^38^^,^^60^ both of which demonstrated similar results. Fruits and vegetables are rich in dietary fiber, vitamins, minerals, and phytochemicals, which help reduce the risk of cardiovascular disease and obesity, while also offering protection against chronic diseases, oxidative stress, and inflammation.^61^^,^^62^

Although the peer-led interventions analyzed in this review were largely successful in improving dietary intake, their effectiveness was not universal. The study by Sharif Ishak and colleagues^49^ found no significant difference in dietary intake between the intervention and control groups. Several factors may have contributed to this outcome. First, the intervention’s short duration of only 16 weeks—one of the briefest in the review—likely limited the time available for sustained behavior change, as longer interventions generally yielded more positive results. Second, the lack of booster sessions may have hindered the reinforcement of new behaviors, which are critical for maintaining dietary changes beyond initial exposure. This gap is particularly important, as other studies in the review with reinforcement mechanisms (eg, follow-up sessions) demonstrated more significant and sustained outcomes.

Furthermore, baseline differences between the intervention and control groups in terms of age, sex, and knowledge score could have skewed the results, suggesting the need for better baseline matching or statistical adjustments to control for these variables. Finally, the small sample size used in the study likely reduced its statistical power, making it difficult to detect meaningful changes in dietary practices. These limitations highlight that while peer-led interventions are promising, their success depends heavily on study design factors such as intervention duration, reinforcement strategies, and sample size.

### Dietary Knowledge

This systematic review showed that peer-led interventions were effective in increasing knowledge about healthy dietary behavior, which consists of understanding the importance of nutrients, balanced diets, and healthy eating habits. These findings are supported by 2 different systematic reviews performed to assess the effect of peer nutrition education, both built on primary studies conducted in the United States and Canada, which highlighted the improved knowledge about healthy dietary behaviors.^7^^,^^63^ The literature suggests that higher levels of knowledge of a healthy diet are positively correlated with consuming more fruits, dairy, protein, and whole grains, which are essential for meeting dietary recommendations.^64^ Additionally, individuals with better knowledge of nutrition tend to make healthier food choices across all food categories.^65^ Increasing knowledge and improving an individual’s belief in their ability to change their habits can help in overcoming significant obstacles to adopting healthier choices. Thus, promoting awareness about healthy dietary behavior through public campaigns can be an effective approach to encouraging healthier eating habits among adolescents.

### Attitude and Self-Esteem

In this systematic review, most studies found that peer-led interventions can improve the attitudes of adolescents toward healthy dietary behavior and their self-esteem with the only exception being the study by Sharif Ishak and colleagues.^49^ A systematic review by Nelson and Nickols-Richardson^63^ supported this finding that peer nutrition education enhanced attitudes and self-efficacy related to healthy eating. Decisions and choices regarding healthy food are significantly influenced by one’s attitude toward healthy food.^66^ When individuals have a positive attitude toward healthy foods, they are more likely to perceive these foods as desirable, enjoyable, and satisfying.^67^ This, in turn, can increase their motivation to choose these foods over less healthy options. Thus, a favorable attitude, although not sufficient cause, helps in improving dietary intake. Likewise, improving self-esteem in adolescents has been linked to better dietary behavior, overall health, and educational outcomes.^68^^,^^69^ It is important to note, however, that the study by Sharif Ishak and colleagues^49^ found no significant difference in attitude scores between the intervention and control groups, possibly due to the factors mentioned earlier in the dietary intake section.

There is existing evidence that shows low self-esteem is associated with poor dietary choices, such as higher consumption of unhealthy foods and lower consumption of fruits and vegetables. Conversely, higher self-esteem is associated with healthier dietary patterns and a lower risk of obesity.^69^ Thus, it is important to improve the self-esteem of adolescents to improve overall dietary behavior.

The findings from this review indicate that peer-led interventions can positively influence adolescent dietary behaviors to some extent in LMICs, but their success is often tied to multiple factors.

In this review, the interventions that applied behavior change theories demonstrated more consistent outcomes than the interventions that did not.^44^^,^^50^ This finding suggests that grounding interventions in psychological theories offer stronger mechanisms for sustaining behavior change. By helping to identify key determinants, such as motivation, social context, and environmental influences,^70^ theories provide a structured foundation for planning effective interventions. In contrast, interventions without a theoretical framework lack this crucial component.

The success of multicomponent interventions, such as those involving community or family engagement, and environmental restructuring highlights the importance of a holistic approach. For example, environmental restructuring strategies—such as reorganizing school canteens and establishment of school media—created broader support systems that reinforced dietary changes, leading to long-lasting effects.^45^^,^^46^^,^^51^ Likewise, the involvement of family and community established wider support networks that strengthened dietary changes, resulting in lasting effects.^46^^,^^51^ These results underscore the supposition that effective peer-led interventions should not only focus on individual knowledge but also modify the broader social and environmental context in which adolescents make food choices.

Additionally, the duration of the intervention plays a critical role in its effectiveness. Longer interventions were demonstrated to be more effective in leading to behavior changes.^45^^,^^46^^,^^48^^,^^50^ Extended intervention periods provided more opportunities for reinforcement, behavior modeling, and habit formation, contributing to long-term improvements in dietary behavior.^71^ This underscores the importance of giving adolescents ample time to internalize new dietary habits and overcome environmental or social barriers to change.

### Gaps in Intervention Design

This systematic review revealed a handful of gaps in the conduct of interventions in the included studies. First, very few studies used a theoretical foundation to design or implement their intervention. Second, none of the interventions we examined involved students in the planning and design. Involving adolescents in the planning process could have resulted in better outcomes^72^ by aligning the interventions with the needs of the participants and addressing issues from their perspective. Third, there is strong evidence showing that peer-led nutritional interventions that demonstrated significant changes in dietary behavior consisted of several components, such as longer follow-up time, the introduction of nutrition education into the regular curriculum, the involvement of school staff and teachers in facilitating the programs, parental engagement, and the utilization of theoretical frameworks to guide the development of the intervention.^63^^,^^73^^,^^74^ Individual studies included in the present systematic review lacked one or more of these features.

### Methodological Strengths

This systematic review has noteworthy methodological strengths. By incorporating studies from various LMICs, this review provides a wide geographical perspective on the effectiveness of peer-led interventions. Additionally, all articles included in this systematic review reported having a control group. These control groups were typically composed of comparable youth of the same age from different schools, allowing for the inference of cause and effect. The fact that there were no cases where members of the control group attended the same school as members of the intervention group prevented the diffusion of the intervention to nonparticipating controls. These circumstances in turn prevented any underestimation of the intervention’s actual impact.

### Limitations

This systematic review has several limitations that should be considered when interpreting the results. One of the primary limitations is the relatively small number of studies included. The limited number of studies increases the risk of bias, particularly the overrepresentation of certain regions or types of interventions. This limitation introduces the possibility that the review’s conclusions are disproportionately influenced by specific cultural or logistical factors that may not be present in other LMICs.

Furthermore, there was significant heterogeneity among the included studies in terms of intervention approaches, durations, and outcome measures. This variation made it challenging to compare the results across studies and attempt to conduct a meta-analysis. As a result, a narrative synthesis was used to integrate the findings, but the wide diversity in study designs and contexts introduced uncertainty regarding which components of the interventions were universally effective. Another key limitation was the lack of long-term follow-up in most studies. Only 2 studies measured outcomes at 3 time points, while the others had relatively short follow-up periods. This limitation restricted our ability to assess the sustainability of the effects of the interventions. While short-term improvements in dietary intake and knowledge were documented, it remains unclear whether these changes were maintained over time.

Finally, the geographical and cultural differences among the included studies add further complexity to the interpretation of the findings. The studies were conducted in diverse regions with varying cultural and socioeconomic contexts, which may have influenced the effectiveness of peer-led interventions. Due to these contextual differences, the success of an intervention in 1 region may not necessarily translate to another. Therefore, the findings of this review should be interpreted with caution when applied to regions outside the specific contexts of the included studies. Policymakers and practitioners should consider local contexts and cultural factors before implementing these interventions at scale.

## CONCLUSION

Based on the key findings of this systematic review, we came across the following important points. First, peer-led, school-based, interventions are effective in improving the dietary behavior of adolescents. Second, integrated and multicomponent interventions are more effective than single-component interventions. Third, interventions with longer durations demonstrated greater improvements in participants compared to interventions with shorter durations. Fourth, while the review focused on programs in LMICs, the populations studied exhibited diversity in age, heterogeneity, and ethnicity, suggesting that peer-led programs can be adapted to various target audiences.

Based on the key findings and conclusions of the systematic review, we propose the following important recommendations. First, future research should focus on identifying the key components that make peer-led interventions successful, particularly the most effective delivery methods and frequency of delivery. Adopting a multicomponent approach is also encouraged to boost intervention effectiveness. Additionally, grounding interventions in a theoretical framework may strengthen behavior change outcomes. Behavioral theories provide structured guidance to the implementation of interventions that could enhance the dietary behavior of adolescents.

Cultural and contextual customization is equally important; interventions should be tailored to the unique characteristics of the target population. Involving community members can help ensure that interventions are culturally relevant and contextually appropriate, further boosting their impact. Involving adolescents in the planning and design of interventions is also recommended to align interventions more closely with adolescent needs and perspectives, potentially fostering more relevant and effective adolescent-centered interventions.

Longer follow-up periods should be included in future studies to assess the sustainability of behavior changes over time, as sustained impact is critical for long-term health improvements. Finally, research should address gaps in under-researched regions to provide a more balanced understanding of the global effectiveness of peer-led interventions across LMICs. Expanding the evidence base in these areas will contribute to more comprehensive, regionally adaptable health strategies for adolescents.

## Supplementary Material

nuaf037_Supplementary_Data

## Data Availability

All data are included in the manuscript, with additional extracted data in Excel format available upon reasonable request.
